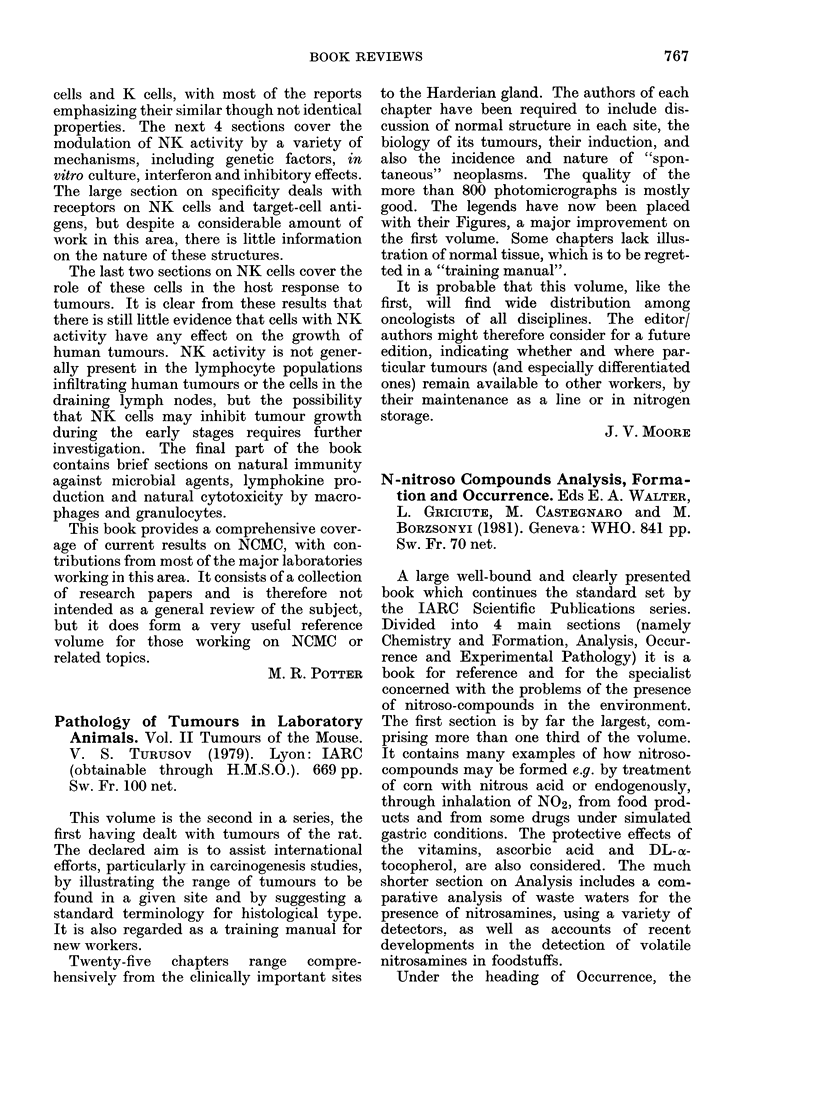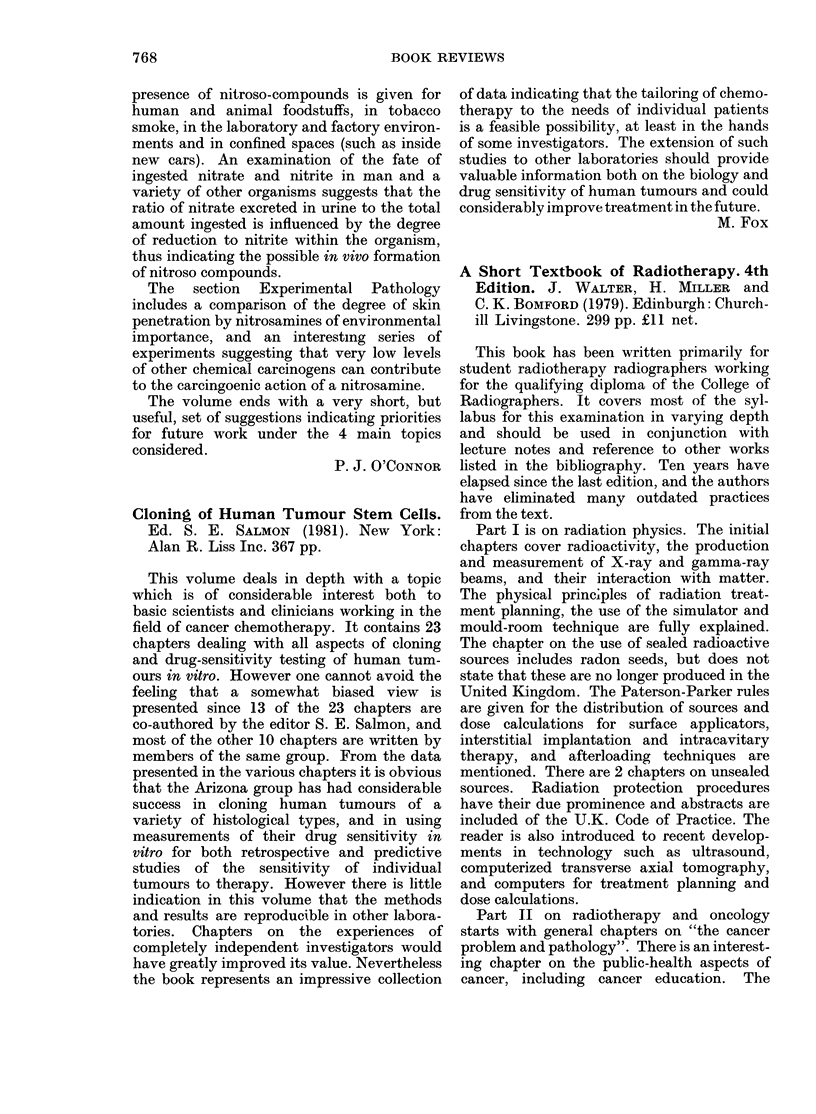# N-nitroso Compounds Analysis, Formation and Occurrence

**Published:** 1981-11

**Authors:** P. J. O'Connor


**N-nitroso Compounds Analysis, Formation and Occurrence.** Eds E. A. WALTER, L. GRICIUTE, M. CASTEGNARO and M. BORZSONYI (1981). Geneva: WHO. 841 pp. Sw. Fr. 70 net.

A large well-bound and clearly presented book which continues the standard set by the IARC Scientific Publications series. Divided into 4 main sections (namely Chemistry and Formation, Analysis, Occurrence and Experimental Pathology) it is a book for reference and for the specialist concerned with the problems of the presence of nitroso-compounds in the environment. The first section is by far the largest, comprising more than one third of the volume. It contains many examples of how nitroso-compounds may be formed *e.g.* by treatment of corn with nitrous acid or endogenously, through inhalation of $NO_2$, from food products and from some drugs under simulated gastric conditions. The protective effects of the vitamins, ascorbic acid and DL-$\alpha$-tocopherol, are also considered. The much shorter section on Analysis includes a comparative analysis of waste waters for the presence of nitrosamines, using a variety of detectors, as well as accounts of recent developments in the detection of volatile nitrosamines in foodstuffs.

Under the heading of Occurrence, the presence of nitroso-compounds is given for human and animal foodstuffs, in tobacco smoke, in the laboratory and factory environments and in confined spaces (such as inside new cars). An examination of the fate of ingested nitrate and nitrite in man and a variety of other organisms suggests that the ratio of nitrate excreted in urine to the total amount ingested is influenced by the degree of reduction to nitrite within the organism, thus indicating the possible *in vivo* formation of nitroso compounds.

The section Experimental Pathology includes a comparison of the degree of skin penetration by nitrosamines of environmental importance, and an interesting series of experiments suggesting that very low levels of other chemical carcinogens can contribute to the carcingoenic action of a nitrosamine.

The volume ends with a very short, but useful, set of suggestions indicating priorities for future work under the 4 main topics considered.

P. J. O'Connor